# Next-generation gene catalogues and genomics tools focused on forestry research

**DOI:** 10.1186/1753-6561-5-S7-P174

**Published:** 2011-09-13

**Authors:** Philippe Rigault

**Affiliations:** 1 Gydle Inc.,1363 av. Maguire, Québec, QC G1T 1Z2, Canada

## 

Gydle is developing reference gene catalogues and genomics resources targeted at forestry research, leveraging its unique bioinformatics expertise in next-generation sequencing.

Next-generation sequencing technologies have revolutionized the field of genomics and have reached the capacity to generate enough quality data to sequence and assemble thousands of genomes and gene catalogues per year. These extraordinary opportunities are currently limited by challenges in data analysis which hinder the translation from raw sequencing data to applications in research and in the field. There is an enormous gap between an instrument capacity to produce millions of reads per day and the ability to analyze and translate quickly this data into usable information and value-added sets for genomics research. One particular limitation stems from the current state of assemblers which produce crude contigs and scaffolds that need considerable editing in order to obtain contiguous, gene-oriented, full-length, artifact-free annotated sequences desirable for the production of quality tools for genomics research. Researchers also lack tools to rapidly integrate and visualize their own data and collaborate in a user-friendly way. Finally, the ability to produce gene catalogues for thousands of samples calls for a new generation of tools to conduct large-scale comparative genomics studies between and within species.

Gydle has developed a complete suite of proprietary bioinformatics tools to address many of the challenges associated with next-gen sequence analysis. A survey of these tools will be presented, which include innovative raw data filtering, rapid and accurate DNA alignment and gene-oriented assembly software, automated and interactive visualization tools for sequence correction and annotation. These tools enable users to process from start to finish a typical next-gen sequencing run in a user-friendly way in a matter of minutes to hours instead of weeks. Users can then visualize their results, edit sequence assemblies and annotations and identify features such as sample-specific expression patterns, SNPs, unspliced introns and alternative splicing events, handling information from hundreds of millions of reads on their workstation or laptop computer. We intend to make these bioinformatics tools available commercially in the near future.

Applying these bioinformatics tools, we combined sequencing data generated by Gydle with publicly available data to produce curated reference gene catalogues for species of interest to the forestry industry, which currently 35 tree species including 7 pinus, 3 picea, 3 eucalyptus, 2 poplar and 5 oak species. Gydle gene catalogues also include over 50 other plant species of agricultural and medicinal interest as well as gene catalogues for species ecologically related to trees such as insects, endophytes, fungi, and microbiota. We intend to make these catalogues available commercially in addition to offering the possibility for researchers to integrate their own data with these catalogues privately.

We used these reference genes catalogues to produce species-specific and cross-species value-added genomics resources such as:

- reference gene sequences covering biochemical pathways involvedin growth, wood formation, climate adaptation, biofuel production, and secondary metabolites of interest to pest resistance and human health

- curated amino-acid databases to improve proteomic and metabolomic identifications

- SNP sets and designs of high-throughput genotyping assays such as Illumina GoldeGate/Infinium for marker discovery and genomic selection.

- reference sets and tools for gene expression studies by RNA-seq and microarrays.

Examples of gene catalogues, cross-species analysis and value-added sets will be presented.

